# The Significance of Type-I Interferons in the Pathogenesis and Therapy of Human Immunodeficiency Virus 1 Infection

**DOI:** 10.3389/fimmu.2017.01431

**Published:** 2017-11-01

**Authors:** Bowen Wang, Wen Kang, Jiahui Zuo, Wenzhen Kang, Yongtao Sun

**Affiliations:** ^1^Department of Infectious Diseases, Tangdu Hospital, The Fourth Military Medical University, Xi’an, China; ^2^Clinical Laboratory, Tangdu Hospital, The Fourth Military Medical University, Xi’an, China

**Keywords:** type 1 interferons, human immunodificiency virus 1, pathogenesis, immunocytes, immunotherapy

## Abstract

Type-I interferons (IFN-I) are a widely expressed family that could promote antivirus immunity in the process of pathogens invasion. In a human immunodeficiency virus 1 (HIV-1)-infected individual, the production of IFN-I can be detected as early as the acute phase and will persist throughout the course of infection. However, sustained stimulation of immune system by IFN-I also contributes greatly to host-mediated immunopathology and diseases progression. Although the protective effects of IFN-I in the acute phase of HIV-1 infection have been observed, more studies recently focus on their detrimental role in the chronic stage. Inhibition of IFN-I signaling may reverse HIV-1-induced immune hyperactivation and furthermore reduce HIV-1 reservoirs, which suggest this strategy may provide a potential way to enhance the therapeutic effect of antiretroviral therapy. Therefore, we review the role of IFN-I in HIV-1 progression, their effects on different immunocytes, and therapeutic prospects targeting the IFN-I system.

## Introduction

Human immunodeficiency virus 1 (HIV-1) is a highly pathogenic retrovirus that causes immune system degeneration (1). In the past 30 years, antiretroviral therapy has achieved considerable advances. However, despite remarkable scientific achievements in HIV-1 diagnosis and treatment, acquired immune deficiency syndrome (AIDS) still prevails and there are estimated 35 million people worldwide living with HIV-1 infection or AIDS (2, 3). The innate immune system, a significant alarm system in our body, has been caught more attention on resisting foreigner pathogens in recent years (4–6). One of the key effector molecules in innate system is interferons (IFNs), which rapidly respond to virus infection by a broadly, non-specific manner.

Interferons are classified into three groups based on the structure of their receptors: type I (IFN-α, IFN-β, IFN-κ, IFN-δ, IFN-ε, IFN-τ, IFN-ω, and IFN-ζ), type II (IFN-γ), type III (IFN-λ1, IFN-λ2, and IFN-λ3). Among these three types, IFN-I bind to a cell surface receptor complex known as the IFN-α/β receptor (IFNAR), which consists of IFNAR1 and IFNAR2 chains (7). Contrary to the limited expression of type II and type III interferons receptor, IFNAR is widely expressed on almost all kinds of immunocytes and epithelial tissue (5, 8, 9), suggesting that IFN-I have an extensive influence and are able to arouse quick activation of the whole immune system.

Innate immune responses mainly derive from the recognition of viral pathogen-associated molecular patterns (PAMPs) by host pattern recognition receptors (PPRs) such as toll-like receptors (TLRs), retinoic acid-inducible gene (RIG)-I-like receptors (RLRs), and other DNA-sensing receptors (10–12). Upon sensing PAMPs, several downstream signal molecules and transcriptional factors will be recruited, subsequently production of IFNs, especially IFN-I, is stimulated. At the earliest stage of acute infection, the production of IFN-I and other inflammatory cytokines is an essential event to determine the rate of virus replication and spreads (11, 13, 14). Unfortunately, this response is usually ineffective to suppress HIV-1 activity, due to the ability of this virus to hijack host immune system and evade the IFN-mediated antiviral activities (4). Moreover, the persistent IFN-I secretion greatly disturbs the immune homeostasis, contributing to immune activation-dependent disease progression (15, 16).

The administration of IFN-I, especially IFN-α, as monotherapy or an adjunct to combined antiretroviral therapy (cART), has been intensively reported (17), but the results varied greatly. In addition, as the continuous IFN-I production impedes immune recovery and enhances T cells exhaustion, IFN-I blockade may provide another strategy to weaken the virus-induced immune hyperactivation in the chronic HIV-1 infection (18, 19). Actually, inhibition of IFN-I system is likely to be an efficient way to reverse excessively elevated IFN-I signaling and rescue specific anti-HIV-1 immunity (20). Concerning the extensive impact of IFN-I system, it is unclear whether this method to shut off the IFN-I system is beneficial to pathogenesis or merely a secondary effect.

In this review, we will discuss the stimulation of IFN-I by sensing viral pathogens after HIV-1 infection, the antiviral/immunomodulatory activities of IFN-I on different immunocytes, and the manipulation of IFN-I system as a therapeutic strategy *in vivo*.

## The Induction of IFN-I in the Process of HIV-1 Infection

### Recognition of HIV-1 by PRRs in Innate Immune System

At the beginning of infection, HIV infects immunocytes such as dendritic cells, macrophages and CD4^+^ T cells in the human intestinal mucosal. In this process, virus can be rapidly recognized by innate immune system through a series of complex mechanisms as follows. The initial sensing of HIV-1 is mediated by PRRs. There are four classes of PRRs family have been identified, including transmembrane protein as TLRs and C-type lectin receptors, as well as cytoplasmic proteins as RLRs and NOD-like receptors (21). They recognize conserved structures of HIV-1 nucleic acid, which is called PAMP. The interaction between PAMP and PRRs will result in different level of immunocytes activation. And evidence has been found that plasmacytoid dendritic cells (pDCs) produce the highest level of IFN-I once sensing PAMP, whereas the production of IFN-I is barely detectable in other immune cells (22).

**Figure 1:**
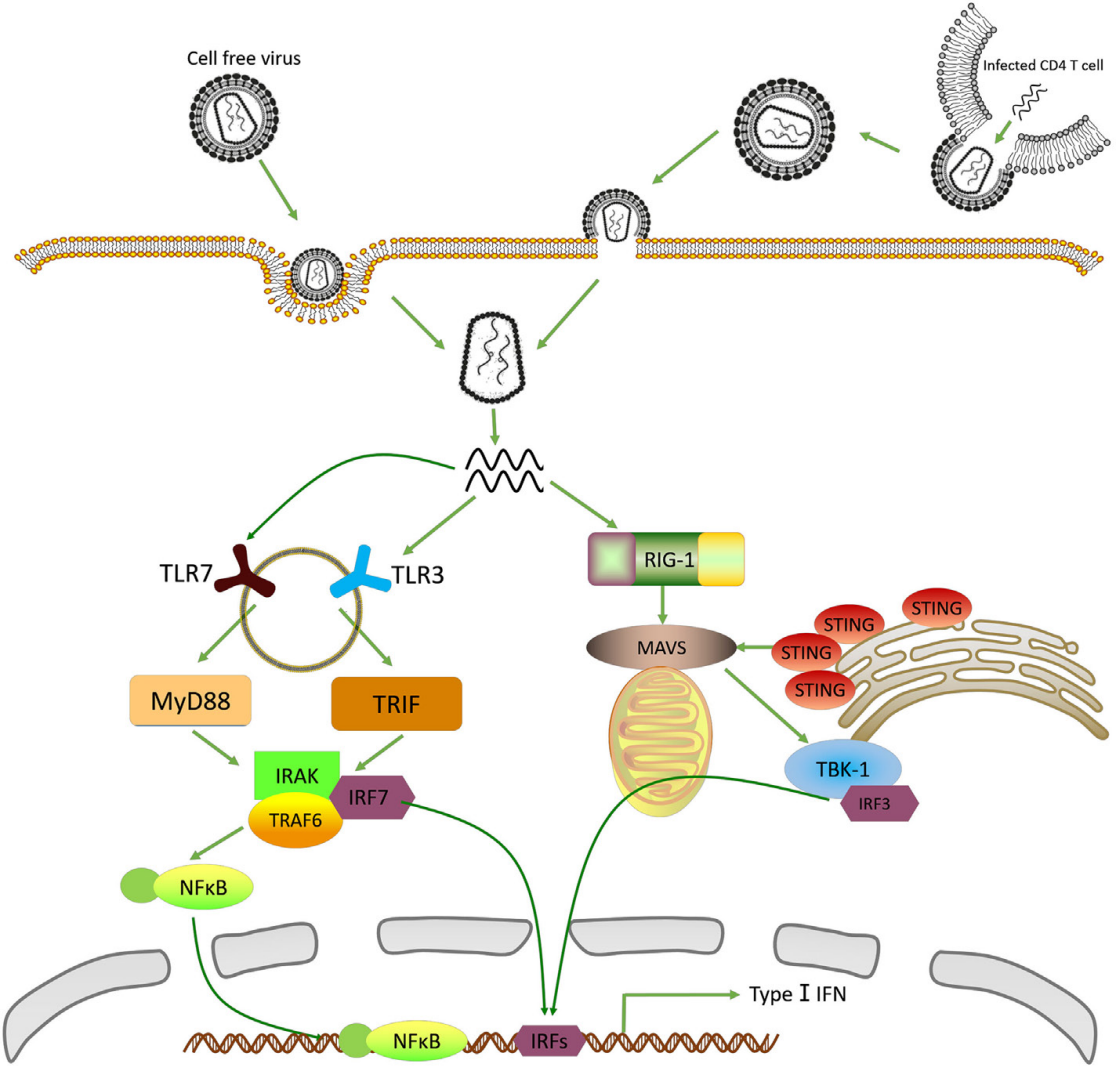
Recognition of human immunodeficiency virus 1 (HIV-1) by innate immune system. In plasmacytoid dendritic cells (pDCs), cell-free HIV is taken up through endocytosis while cell-associated virus enters into pDCs by fusion and endocytosis. The single-strand RNA (ssRNA) released from virus is recognized by TLR3 and TLR7. Then the activated toll-like receptors (TLRs) stimulate MyD88 and TRIF signal pathway, recruit NF-κB and interferon-regulatory factor (IRF)-7, respectively, to trigger type-I interferons (IFN-I) production. In macrophages and CD4^+^ T cells, HIV-1 enters cells mainly through fusion and endocytosis. But the ssRNA is detected by retinoic acid-inducible gene (RIG)-I, which stimulates mitochondrial antiviral signaling protein (MAVS)–IRF-3 dependent pathway, and next moderately induces the expression of IFN-I.

HIV *in vivo* can be divided into two types: cell free virus and cell-associated virus. The former is the virus infected directly from outside; while the latter is produced through viral RNA reverse transcription, integration and packaging in infected CD4^+^ T cells (Figure 1). However, they enter pDCs through separate pathways. Cell free virus are taken up by pDCs through endocytosis mediated by envelope–CD4 interaction (23, 24), while cell-associated virus enter pDCs by fusion or endocytosis (14). Although cell-associated viruses are at a high level *in vivo*, they are a less potent inducer of IFN-I than cell free virus (25). One of the possible reasons is that most of cell-associated viruses are defective viruses which are not able to induce effective immune responses.

Plasmacytoid dendritic cells highly express TLR7, which greatly enhances their ability to produce IFN-I up to 1,000-fold more than other cell types’ response to HIV-1 infection (26). In the cytosol of pDCs, HIV nucleic acid is presented to TLR7 located in endosomes, which is transferred from the endoplasmic reticulum (ER) to endosome *via* polytopic membrane protein UNC93B1 and heat shock protein gp96 (27, 28). After the formation of endosomes, TLR7 rapidly catches up with single-strand RNA (ssRNA) (29). But the specific character of these ssRNA has not been identified. Moreover, TLR3 is the other essential TLR expressed in pDCs, which detects both ssRNA and double strands RNA (dsRNA) (30, 31). Similarly, TLR3 also transferred from ER to endosome. In mature endosome, activated TLR7 and TLR3 by pathogenic nucleic acid is phosphorylated by tyrosine kinase-Src (32, 33). Although TLR7 and TLR3 are both explicitly expressed in pDCs, TLR7 plays a more important role in recognition of pathogenic nucleic acid and stimulation of IFN-I in response to HIV-1 infection (34).

Although ways of HIV-1 entering into macrophage and CD4^+^ T cells are similar to that entering into pDCs, the recognition of HIV nucleic acid by these cells are completely different. In these cells, viral RNA is detected by RIG-I, a cytosolic receptor, without the generation of endosomes. RIG-I is a member of DExD/H box RNA helicases family. The crystal structure of RIG-I can be divided into three distinct domains: N-terminal region consisting of caspase activation and recruitment domains (CARD) to trigger IFN-I secretion; a central DExD/H box RNA helicase domain binding to specific RNA; as well as C-terminal repressor domain (35, 36). Just because of the special structure of RIG-I, PAMP of RNA virus whose sequence is marked with 5′ triphosphorylated (5′ppp) ends could be well recognized (35). It is by 5′ppp marks that RIG-I distinguishes exogenous RNA from their own (37). Various studies confirm that RIG-I^−/−^ mice become more susceptible to RNA virus infection (35, 38, 39). Furthermore, stimulating the RIG-I pathway by retinoic acid effectively reactivates HIV reservoirs and promotes apoptosis of these infected cells, leading to the enhancement of innate immune system to eliminate latent reservoirs (40).

### Interferon-Regulatory Factors (IRFs) Regulating the Production of IFN-I

Interferon-regulatory factors are a member of transcription factors that place in the central position of innate immune responses. Actually, they play a crucial role in bridging PRRs and the induction of IFN-I in gene-regulatory network (4). IRFs family consist nine members: IRF 1–9. The common of these transcriptional factors is that they all contain a conserved DNA-binding domain to recognize DNA sequences known as IFN-stimulated response element (41). Especially IRF-3 and IRF-7, they are main regulators in producing IFN-I among pDCs, macrophages and CD4^+^ T cells after the interaction of PAMP and PPRs.

Interferon-regulatory factor-7 has attracted much attention for its function in pDCs. Upon recognition of HIV-1 ssRNA by TLR7 and TLR3 in endosome, a complex including IRF-7, IRAK, TRAF6, and other proteins are rapidly recruited (42, 43). However, the complex is engaged by these two TLR through distinct pathways: TLR7 is mediated by a MyD88-dependent manner whereas TLR3 by TRIF (44, 45). The establishment of complex drives the phosphorylation of IRF-7 by IRAK1 and IKKα. Then phosphorylated IRF-7 translocates from cytoplasm into nucleus, attach to the promoter of IFN-I and increase their expression (42, 46). At the same time, the complex activates NF-κB by a MyD88-TRAF6 dependent pathway, which further stimulates the production of IFN-1. Notably, phosphorylated IRF-7 can form a dimer (a homodimer or a heterodimer with IRF-7), which stimulates the activity of histone-acetyltransferase to loosen chromatin structure and facilitate more efficient transcription of IFN-I (47).

The other significant IRFs that have been intensively studied in HIV infection is IRF-3. Similar to IRF-7, IRF-3 also resides in the cytosol. However, the recruitment of IRF-3 follows the interaction between viral RNA and RIG-I in macrophage and CD4^+^ T cells (37). During viral infection, 5′ppp RNA PAMPs bind to R-terminal region of RIG-I, which cause the release of CARD to trigger CARD-depended interaction with mitochondrial antiviral signaling protein (MAVS) that is located on the outer mitochondrial membrane (48, 49). The activation of MAVS strongly catalyzes 2,3′-guanosine-adenosine monophosphate (cGAMP), which is the paramount agonist of stimulator of interferon genes (STING). STING initially aggregate around the MAVS, then stimulate the downstream signaling cascades that involve multiple kinases and finally lead to the phosphorylation of IRF-3 (50). Following behind phosphorylation, IRF-3 shares the similar mechanism of facilitating the transcription of IFN-I as IRF-7. Importantly, recent advances show that reverse transcribed HIV-DNA but not its RNA induces IRF-3 activation and IFN-I production depend on cGAMP–STING–IFI16 pathway in macrophage (51–53). In addition, the polyglutamine binding protein 1 (PQBP1) is recently identified as the co-receptor of HIV-DNA to trigger cell-autonomous antiviral responses (54). Therefore, PQBP1 as an immune regulator provides a pharmacological target to improve the efficiencies of HIV medicine. However, sensing of HIV-DNA by IFI16 cannot induce IFN-I production in CD4^+^ T cells, resulting the HIV evasion from innate immune system and formation of HIV reservoirs (55).

Interestingly, IRF-3 functions at the initial transcription of IFN-I gene. Whereas IRF-7, the upregulation of which also need the stimulation by IFN-I itself, is involved in the late phase of IFN-I gene induction. That is to say, the induction of IFN-I by IRF-3 is mediated by a two-step activation, which forms a positive-feedback-loop (56). It is likely another reason that pDCs produce the highest level of IFN-I as is mentioned earlier.

## Effects of IFN-I on Immunocytes in Chronic HIV-1 Infection

Unlike the effective response in other infectious diseases, IFN-I in HIV-1 infection becomes rapidly dysfunctional and unable to purge the virus finally. Inversely, as the widely expression of IFNAR, prolonged virus replication and sustained stimulation of IFN-I progressively induce a generalized immune activation, injured inflammation, as well as T cell exhaustion (2, 46). Moreover, a strong correlation between the levels of IFN-I and disease progression has already been observed. In the models of simian immunodeficiency virus (SIV) infection, the common character of long time non-progression macaques is that they can induce rapid and transient high levels of IFN-I but declines in the chronic phase (19, 57, 58). Though the administration of cART can effectively suppress the replication of HIV-1, it cannot completely reverse the immune hyperactivation caused by IFN-I (59, 60). In the lymphocytic choriomeningitis virus (LCMV) mouse models, persistent production of IFN-I exacerbates CD4^+^ T cell exhaustion and is detrimental to its antiviral response (61–63). Other researches also indicate that chronic infection of LCMV induces inhibitory molecules expression and apoptosis of Treg (64, 65). These studies suggest that compare to a positive role of IFN-I on restricting virus spread and replication at the acute phase of infection, IFN-I tend to exert a negative effect on different immunocytes in chronic HIV-1 infection.

Plasmacytoid dendritic cells are a special dendritic cell subset that produces a large amount of IFN-I in the process of HIV-1 infection. With the progression of HIV-1 infection, pDCs gradually decrease in blood while accumulate in lymph nodes. pDCs from these lymph nodes secrete higher titer of IFN-α spontaneously but not express co-stimulatory molecular (66). These nonfunctional cells are continuously produce IFN-I but cannot develop into mature antigen presentation cells. Instead, the redistributed pDCs increase staining of Annexin V and thus exhibit apoptosis (67). Even for cART treatment patients, the frequency and function of pDCs in peripheral blood is decline and accumulate in gut-associated lymphatic tissue (68). Moreover, in the chronic phase of HIV-1 infection, the excessive IFN-I may result in the dysregulated activation and even depletion in pDCs. In SIV models, a negative correlation between the decline of circulating pDCs and overexpression of IFN-I are observed during pathogenic SIV infection of macaques, but not in natural ones (69). Furthermore, pDCs with the upregulation of β7-integrin and CD103 are aggregated to the colorectum in chronic HIV-1 infected patients, which facilitates much more production of IFN-I (70, 71). Unfortunately, the amount of IFN-I produced by these lymphatic tissues is much more than what organisms really need to fight infection (71). Furthermore, these redundant IFN-I will lead to the activation of innate immune system and conversely damage the normal function of pDCs. However, the specific mechanism how IFN-I interacts with pDCs has not been clearly elucidated. There are two possible reasons for this phenomenon: On the one hand, pDCs that are persistently stimulated by IFN-I express low levels of migration receptors and regulatory factors, such as CCR7, CD40, and CD86. These pDCs produce IFN-I since pathogenic nucleic acid traffics to the endosome (72, 73), which induces apoptosis to these out-of-control cells mediated by the TNF-related apoptosis-inducing ligand (TRAIL) (74). On the other hand, IFN-I activates the non-canonical NF-κB signaling in pDCs. This pathway will promote the expression of indoleamine 2,3-dioxygenase, the most essential factor to gather regulatory T cells (Treg) (75). These pDCs-induced Treg intensively inhibit the maturation of pDCs through the engagement of cytotoxic T-lymphocyte antigen (CTLA)-4 and PD-1 on these activated pDCs (6).

Natural killer (NK) cells play a crucial role in innate immune system that act as the first line to defense HIV-1. First, the interaction between killer immunoglobulin-like receptors expressed on the surface of NK cells with their cognate HLA ligands sets a guarantee recognize specific HIV-derived peptides and eliminate HIV-1 infected cells (76, 77). Second, antibody-dependent cellular cytotoxicity (ADCC) is the other way of NK cells to control HIV-1 infection. Notably, ADCC activity was associated with the modest protective efficacy in the RV144 HIV vaccine trial (78). Moreover, new data show that the levels of NK cells activation is tightly associated with HIV-1 virological suppression in patients receiving cART (79). Patients who initiate ART early during infection obtain the improvement of cytotoxic function of the NK cells while decline of the levels of ADCC mediating antibodies (80, 81). These data suggest that NK cells. However, during the whole phase of HIV-1 infection, the relation between NK cells and IFN-I is complicated. In the acute infection, IFN-I has been observed to promote NK cells survival, expansion, maturation, activation and enhance their cytotoxic activity against virus (82, 83). In IFNAR^−/−^ mice, there are barely detectable mature NK cells in peripheral blood (84). Identically, impaired cytotoxicity ability as well as a loss of highly activated subset of NK cells has been found in rapid progressors (85). But in the chronic HIV-1 infection, IFN-I may greatly disturb the normal function of NK cells. First, despite viral control well, NK cells are consistently activated in the chronic HIV-1 infection after the establishment of reservoirs. In the presence of high level of IFN-I, activated NK cells attenuate the cytotoxicity of CD8^+^ T cells response to virus infection. As the suppressed function of NK cells and CD8^+^ T cells, HIV escape from ADCC and CTL effect (86). Another research demonstrates that NK cells-depletion mice promoted virus-specific T cells response and contributed to viral control (87). Second, IFN-I disturbs the balance between STAT1 and STAT4, two downstream transcriptional factors of IFNAR in NK cells. In HCV/HIV coinfected patients, high level of IFN-I is relevant to the upregulation of STAT1 while downregulation of STAT4, which increase the expression of perforin induced by interleukin (IL)-12 (88). This mechanism further prompts the nonfunctional activation of NK cells. Thirdly, IFN-I decreases the production of IFN-γ through upregulating the expression of IL-10 and PD-L1 in NK cells (89), which further weaken their cytotoxic effects.

It is well established that HIV-infected patients maintain persistently high circulating CD8^+^ T cells number, in spit of many years of therapy (90). The CD4/CD8 ratio often fails to become normal despite CD4 count normalization. Notably, new data showed that the majority of CD8^+^ T proliferation and activation was induced in an antigen-independent manner (91). The great disturbing of T cell homeostasis makes the immune system dysfunctional and exhausted in the chronic HIV-1 infection. It is intensely investigated that IFN-I is responsible for the expansion of CD8^+^ T cells. On the one hand, IFN-I has been shown to induce memory CD8^+^ T cells proliferation and differentiation through bystander effect (92, 93). Thus, it is hypothesis that sustained exposure to IFN-I could contribute to CD8^+^ T cell persistence. On the other hand, during the chronic infection, IFN-I favor the formation of terminally differentiated CD8^+^ T cells that do not renew but enhanced cytotoxic function. This subset skewing likely contributes to the progressive IFN-I-mediated immune dysregulation (61, 89).

As we all known, HIV-1 features as the destroyer of CD4^+^ T cells. It is the death of this kind of cell that propels the late phase of clinical procession, AIDS (2, 26). Although IFN-I could silence T cells to limit viral replication and program cells death to get rid of HIV-1 infection, IFN-I also influence the differentiation of T cells and induce death of HIV-1 uninfected cells *via* bystander effect (92, 94). On the one hand, depending on cytokine environment, naïve CD4^+^ T cells mainly differentiate into Th1, Th2, Treg, and follicular T helper (Tfh) cell populations that have different biological functions (95). Actually, continuously stimulated by IFN-I facilitate naïve CD4^+^ T cells differentiate toward Th2 cells, leading to a severe disproportionality of Th1 and Th2 (96, 97). On the other hand, IFN-I induces apoptosis of uninfected CD4^+^ T cells in peripheral blood and in secondary lymphatic tissue by upregulating TRAIL expression on CD4^+^ T cells, leading to the destruction of lymph node in gastrointestine in the acute phase of HIV-1 infection (98). Recent studies revealed that the death of 95% resting, non-permissive CD4^+^ T cells are caused by caspase-1-mediated pyroptosis, a highly inflammatory form of programmed cell death (99, 100). With the stimulation of IFN-I, the expression of PD-1 is upregulated in exhausted T cell. Instead, blockade of PD-1/PD-L1 pathway helps to restore the function of T cells and decrease the viral load (101). Another studies also show that IFN-I significantly suppress HIV-1-specific CD4^+^ T response, while blockade of IFN-I signaling pathway inactivates immune system, downregulates the expression of negative immune-regulatory factors and maintains the lymphoid structure after chronic LCMV infection (89, 102). In the model of humanized mice, blocking IFNAR rapidly enhances CD4^+^ T recovery and reduces HIV-1 reservoirs (103).

Recently, a special subset of CD4^+^ T cells, Tfh cells, has been intensively reported for its function to induce memory B cells activation, survival, differentiation, as well as assist B cells to produce antigen-specific neutralizing antibodies. At the same time, Tfh cells act as the major CD4^+^ T cells compartment for HIV-1 infection, replication, and long-lived viral reservoirs (104, 105). In the chronic HIV-1 infection, the differentiation of Tfh cells impairs greatly as the repressive effect by excessive production of IFN-I and the activation of signal transducer and activator of transcription 3 (106). But this effect can be partly conversed by blocking the IFNAR in mice (107). Moreover, the population of HIV-specific Tfh cells expand during the chronic phase in patients who have a relatively high level of plasma HIV-RNA and IFN-I, which in turn leads to perturbation of B-cell differentiation, resulting in dysregulation antibody production (108). While in elite controllers, a stronger capacity to induce B cells maturation in Tfh cells is always tightly correlated to the low level of IFN-I (109, 110).

In addition, several studies indicate that the IFN-I also damage the normal function of B cells in the consistent presence of IFN-I. Indeed, IFN-I, especially IFN-α, is one of the most essential factor that contribute to the B-cell hyperactivation and exhaustion in HIV-1 viremic individuals (111–113). The loss of CD21 expression on exhausted B lymphocytes is a reliable marker of HIV-1 disease progression (114). It has been demonstrated that these cells that express low levels of CD21 is associated with the high expression of inhibitory markers as PD-1 and CTLA-4 (115, 116). Interestingly, IFNAR also express highly in these cells, which suggests that IFN-I plays a role on B cells dysfunction (117, 118). Another subpopulation of lymphocytes response to IFN-I is Treg, who work as a dominant role in immunosuppressive function to protect body from unwanted immune responses and maintain the homeostasis of immune system (119). However, the function of Treg is suppressed during the process of HIV-1 infection. When IFN persistently stimulated by IFNAR expressed on Treg, downstream signaling molecules will have impact on the expansion and suppressed function of Treg in turn (120). On the one hand, IFN-I inhibits Treg proliferation through a higher phosphorylation of STAT1 and a lower expression of suppressor of cytokine signaling 1 (121). Notably, this inhibition is tightly correlated with lower frequency of virus-specific CD8^+^ T response and viral clearance (122). On the other hand, chronic HIV-1 infection also leads to the downregulation of Foxp3. Foxp3 appears to act as a master regulator on the development and suppression function of Treg (123, 124). The possible mechanism is that IFN-I induces the secretion of IL-10 and decreases transforming growth factor-β. The overexpression of inhibitory inflammatory factors will competitively inhibit the suppressive function of Treg to rectify the disorder in immune system, which causes dysregulation in Foxp3 (125–127).

Overall, these observations confirm that the detrimental effect of IFN-I on immunocytes is evident during chronic HIV-1 infection. Despite the effective suppression of HIV viral load achieved by cART, it cannot completely reverse the immune hyperactivation and negative function induced by IFN-I. How to take advantage of IFN-I to restrict HIV-1 replication while restore the normal function of immunocytes is a key point we must balance.

## Therapeutic Strategies Targeting IFN-I System *In Vivo*

As is mentioned earlier, although it is apparent for the importance of IFN-I on limiting HIV-1 in the acute phase of infection, dysfunctional IFN-I is more likely to disturb the balance of immune system and be detrimental to the function of pDCs, NK cells, CD4^+^ T cells and Treg in the chronic phase. Considering the complexity of IFN-I system, manipulating this system may have unpredictable consequences *in vivo*. Thus, it is reasonable to use or block this system in the treatment and prevention of HIV infection, but the virological and immunological effects must weigh against the possible adverse events. Nowadays, therapeutic strategies toward IFN-I system consists of two aspects: administration of IFN-I or inhibiting its signaling pathway. Next, we will summarize the related studies about IFN-I system in clinical trials or in HIV-1/SIV/LCMV models.

### Clinical Effects of Administration of IFN-I in HIV-1 Infection

The administration of IFN-I to resist HIV infection undergoes two stages. In the era of pre-cART, IFN-I can be used as a monotherapy on HIV/AIDS. In 1990, a randomized, double-blind clinical trial conducted in 34 HIV-infected patients with the treatment of IFN-α 2b showed that early administration of IFN-I could decrease the frequency of viral isolation and slow the progress of disease despite a few side-effects such as flu-like symptoms and granulocytopenia (128). Another trial fixed attention on patients with AIDS-associated Kaposi’s sarcoma. Although the absolute CD4^+^ T cells count in these patients was relatively low, 50% of them were observed on tumor regression and reduction in HIV after treatment with IFN-α for 12 weeks (129).

However, with the development of cART, IFN-I is more likely to act as an adjunct therapy instead of monotherapy. In recent 10 years, most of trials were designed to treat patients with IFN-α in association with cART, but the results varied greatly (summarized in Table 1). In 2009, more than 200 treatment failure patients were enrolled to receive the administration of IFN-α before optimization of their antiretroviral therapy, finally they were observed a significant decrease in HIV-RNA, but no effect on CD4^+^ T cells count compared with placebo-controlled group (130). Subsequently, several studies carried out in treatment naïve patients, although the majority of these patients had a high level of HIV-RNA at first, administration of IFN-α resulted in a great decline in viral load as well as a transient increase in CD4^+^ T cells count (131, 132). At the same time, IFN-α was added to current treatment of cART, but the results seemed paradoxical: one indicated that IFN-α had a negative effect on disease progression (133), while the other suggested IFN-α decreased HIV reservoirs and delayed virus rebound after treatment interruption (134). Recently, two researches recruited patients who was coinfected with HIV/HCV and received IFN-α in combination with ribavirin, the result of which showed a reduction in integrated HIV-DNA and 2-LTR circular HIV-DNA (135–137). Altogether, administration of exogenous IFN-α intends to decrease HIV-RNA or HIV-DNA transiently in these studies. But the mechanism of IFN-α-induced reduction of HIV-RNA or HIV-DNA remains uncertain. Notably, it is well recognized that IFN-α treatment is related to decreasing CD4^+^ T-cell counts, raising the possibility that reductions of HIV viral load during IFN-α therapy may result from its unspecific lymphocellular toxicity (138). Although IFN-α could decrease the viral burden, adding IFN-α to the current antiretroviral therapy is not likely to enhance T cells reconstitution and improve clinical outcome. Due to the high variability of HIV, the effects of exogenous IFN-α will be rapidly compromised and dysfunctional. Indeed, these IFN-α further disturbs the balance of immune system and even have a negative effect to some extent.

**Table 1:** Studies of IFN-I administration in human.

Participants	Intervention	Conclusions	Reference
261 HIV-infected patients failing current cART treatment with plasma HIV-1-RNA >2,000 copies/mL	0.5, 1.0, 1.5, and 3.0 µg/kg pegylated IFN-α or placebo with current cART × 4 weeks followed with optimized cART × 24 weeks	IFN-α greatly decreases HIV-RNA level No significant changes in CD4^+^ and CD8^+^ T cells count between treatment and control arms	Angel et al. (130)

174 HIV-infected patients without receiving cART with CD4^+^ T cells count ≥500 cells/μL	200 mg/4 h AZT × 52 weeks 1 MIU/day IFN-α 2b with IFN-dose escalation × 52 weeks 200 mg/4 h AZT in combination with 1 MIU/day IFN-α 2b × 52 weeks	In combination with IFN-α greater decreases HIV-RNA level than AZT alone IFN-α transiently increase the CD4^+^ T cells count	Tavel et al. (131)

13 HIV-infected patients without receiving cART with CD4^+^ T cells count ≥300 cells/μL and plasma HIV-1-RNA >5,000 copies/mL	180 μg/week of pegylated IFN-α 2a × 12 weeks	Pegylated IFN-α 2a slightly decreases HIV-RNA and increases in CD4^+^ T cells count	Asmuth et al. (132)

168 HIV-infected patients receiving cART with CD4^+^ T cells count ≥350 cells/μL and plasma HIV-1-RNA <400 copies/mL	1.5 μg/kg/week pegylated IFN-α 2a from day 15 followed by cART interruption to day 8 after each cART resumption	IFN-α greatly decrease the CD4^+^ T cells count and do not prolong the time to treatment resumption	Boué et al. (134)

89 HIV-infected patients without receiving cART	1 μg/kg/week pegylated IFN-α 2a × 14 weeks + cART 1 μg/kg/week pegylated IFN-α 2a × 14 weeks + cART × 36 weeks followed by interruption at week 36, 48, and 60 1 μg/kg/week pegylated IFN-α 2a × 14 weeks + cART × 36 weeks followed by interruption at week 36, 48, and 60 with IFN-α	Viral rebound and HIV-DNA is lower in IFN-α group but no difference after 6-month interruption CD4^+^ T cells count is higher in IFN-α group but also no difference after 6-month interruption	Goujard et al. (133)

23 HIV-infected patients receiving cART with CD4^+^ T cells count >450 cells/μL	180 μg/week of pegylated IFN-α 2a with cART × 5 weeks + pegylated IFN-α 2a with cART interruption × 12 weeks 90 μg/week of pegylated IFN-α 2a with cART × 5 weeks + pegylated IFN-α 2a with cART interruption × 12 weeks	Pegylated IFN-α 2a results in a sustained control of viral replication in 45% of subjects with cART interruption and a significant reduction of integrated HIV-DNA in CD4^+^ T cells	Azzoni et al. (135)

12 HIV/HCV-coinfected patients receiving cART with suppressed HIV-1 viremia	180 μg/week of pegylated IFN-α 2a and ribavirin 500–600 mg twice daily	Approximately twofold decreases of total and integrated HIV-DNA in CD4^+^ T cells during and after IFN-α/ribavirin therapy	Sun et al. (138)

15 HIV/HCV-coinfected patients 17 HIV-infected patients	180 μg/week IFN-α and ribavirin 900 mg twice daily × 48 weeks + cART cART	IFN-α obviously decreases CD4^+^ T cells count and HIV-DNA, especially 2-LTR circular HIV-DNA	Jiao et al. (136)

162 HCV treatment naïve or experienced patients coinfected with HIV-1	ca. 750 mg/8 h telaprevir + 180 μg/week IFN-α and ribavirin 800 mg/day × 18 weeks + cART	Telaprevir and IFN-α decreases CD4^+^ T cells count and three patients had a viral load increase ≥200 copies/mL	Montes et al. (137)

*IFN, interferon; IFN-I, type-I interferons; HIV-1, human immunodeficiency virus 1; cART, combined antiretroviral therapy*.

### Influences of Inhibiting IFN-I Signaling *In Vivo*

Faced with challenge about immune disorder in long-term treatment of cART, more attention is fixed on inhibiting IFN-I signaling pathway to reverse hyperactivation and exhaustion of immune system caused by redundant production of IFN-I. Recent studies *in vivo* about manipulating IFN-I system consists of two aspects: inhibiting TLR to decrease the production of IFN-I and blocking IFNAR to interfere with its signaling (summarized in Table 2).

**Table 2:** Studies of TLR and IFNAR blockade *in vivo*.

Species	Virus	Methods	Conclusions	Reference
Human	HIV-1	13 patients without receiving cART with CD4^+^ T ≥250 cells/μL treated with chloroquine or placebo for 2 months in chronic HIV-1 infection	Significantly reducing CD4^+^ and CD8^+^ T cells activation	Murray et al. (140)

Human	HIV-1	83 patients without receiving cART with CD4^+^ T ≥400 cells/μL treated with hydroxychloroquine or placebo for 48 weeks in chronic HIV-1 infection	Hydroxychloroquine tolerated well No effect on CD8^+^ T cells activation Increasing viral load and declining CD4^+^ T cells count	Paton et al. (141)

Human	HIV-1	19 patients on cART with CD4^+^ T ≤350 cells/μL and undetectable viral load treated with chloroquine in combination with cART for 24 weeks in chronic HIV-1 infection	Chloroquine tolerated well Increasing the level of IFN-α2 production No effect on CD4^+^ and CD8^+^ T cells recovery, T cell activation and inflammation markers in plasma	Routy et al. (142)

Rhesus macaques	SIV	Chloroquine to inhibit TLR7 and TLR9 signaling in acute in acute SIV infection	No changing in the level of cell activation Temporary increasing the expression of interferon-stimulating genes Decreasing CD4^+^ T cells recovery	Vaccari et al. (143)

Rhesus macaques	SIV	IFNAR antagonist to block IFN-α2 activity or exogenous IFN-α treatment in acute SIV infection	Higher viral load and accelerating disease progression whether by administration of IFNAR antagonist or induction of an IFN-tolerate state	Sandler et al. (17)

Mice	LCMV	Anti-IFNAR (MAR1-5A3) and clodronate liposomes in chronic LCMV infection	Preserving the function of virus-specific B cells and accelerating neutralizing antibody production	Moseman et al. (145)

Hu-mice	HIV-1	Using a monoclonal antibody to block IFNAR2 (clone MMHAR-2) in chronic HIV-1 infection	Reversing immune exhaustion IFNAR blockade in combination with cART achieving faster viral suppression and lower HIV-1 reservoirs	Zhen et al. (144)

Hu-mice	HIV-1	Using a monoclonal antibody to block IFNAR1 (extracellular domain and transmembrane domain) in chronic HIV-1 infection	Greatly suppressing aberrant immune activation Reducing the exhaustion of T cells Decreasing HIV-1 reservoirs and delaying virus rebound after cART discontinuation	Cheng et al. (103)

*HIV-1, human immunodeficiency virus 1; IFNAR, IFN-α/β receptor; TLR, toll-like receptor; cART, combined antiretroviral therapy; SIV, simian immunodeficiency virus; LCMV, lymphocytic choriomeningitis virus*.

As is mentioned earlier, pDCs play a significant role in producing IFN-I. It may be an effective way to administrate chloroquine, a novel endosomal inhibitor in blocking TLR7 and TLR9 in pDCs, to reduce IFN-I products (139). Unfortunately, the results of several clinical trials about this strategy seem complicated. First, after receiving chloroquine for 2 months in 13 cART-naïve patients, decreased CD4^+^ and CD8^+^ T cells activation were observed (140). Although the result was encouraging, later research indicated that receiving hydroxychloroquine seemed to be no effect on CD8^+^ T cells activation while hindered the recovery of CD4^+^ T cells (141). Similarly, chloroquine had a negative influence on immunological non-responders (142). Therefore, the impact of reducing IFN-I production through chloroquine is still controversial, which needs further research.

On the other hand, antagonist of IFNAR has been used to specifically block IFN-I signaling in recent years. In 2014, a study showed that IFNAR blockade in the acute SIV infection resulted in increasing viral load, down-expression of antiviral genes and leading to the depletion of CD4^+^ T cells (17). Two studies conducted in the model of hu-mice also found that IFNAR blockade was an effective way to diminish T cells exhaustion and restore immune function in the chronic HIV-1 infection. More importantly, declined in HIV-1 reservoirs was also observed in these researches (103, 143, 144). However, in the model of LCMV, blocking IFNAR can completely reverse the depletion of LCMV-specific B cells and promote the secretion of neutralizing antibody to resist infection (145). These data suggest that IFNAR blockade in combination with cART may provide a potential therapeutic strategy for HIV-1 infection. On account of the complex impact of blocking IFNAR *in vivo*, the assessment of anti-immunological consequences and side-effects must be carried out before widespread implementation.

## Conclusion

Innate immune system provides an immediate defense against pathogens to protect our body from infection by other organisms. As the most important effector molecule, IFN-I is powerful to suppress HIV and stimulate the expression of a bunch of antiviral genes replication at the early phase of infection. Although IFN response seems to be effective, HIV evades the IFN-mediated antiviral activities later since the stimulation of resistance factors. According to the results of clinical trials mentioned earlier, administration of IFN-I may have no benefit on clinical outcome, confirming that it fails to restrict and clear HIV *in vivo*. However, several aspects about this process still require further investigation: in addition to IFN-α and IFN-β, the role of the other subtypes of IFN-I in this process has not been clear; the separate pathways to recognize HIV nucleic acid (ssRNA, dsRNA, or cDNA) needs to be identified in all types of infected cells; and the exact mechanism of HIV escape also have to be clarified. Future studies should address these uncertain questions.

Even if IFN-I do function in the early HIV infection, continuously production promotes immune activation and ultimately exhaustion of immune system in the chronic phase. The detrimental effects of IFN-I on different lymphocyte have been intensely reported. But the regulatory mechanism in different lymphocytes has to be figured out.

Although the long-term clinical results of the administration of exogenous IFN-α seem to be invalid in the acute phase of HIV-1 infection with or without cART, it has been identified that this treatment could reduce the viral load transiently. Future studies should lay emphasis on whether supplementation of IFN-I to cART therapy during chronic infection could further decrease the HIV-1 reservoirs.

Moreover, in animal infectious model, IFN-I blockade strategy contributing to the recovery of T cells and decline in HIV reservoirs have been observed, suggesting that animals obtain benefits from this strategy. Although the results are encouraging, this approach has not been tried in human for its complicate effects. Further proof needs to be provided whether it is effective to decrease the morbidity of AIDS and reduce reservoirs through blocking IFN-I signaling, as this strategy may decrease immune activation whereas increase T cell responses. What is more, if it is possible, clinical trials about effective monoclonal antibody toward IFN-I blockade are expected to conduct in human.

In summary, despite long terms of research, the exact relationship between the production of IFN-I, the pathway of viral evasion, and the induction of pathogenic cellular immunological injury has not been clearly deciphered. Numerous questions remain to answer. A better understanding of the role of IFN-I in HIV pathogenesis will aid in managing this pathway for therapeutic purposes.

## Author Contributions

BW reviewed the mechanisms about the production of IFN-1 in the acute phase of HIV-1 infection; WK provided the data of the clinical trials by administration of IFN-1 in HIV patients; JZ gathered the information of negative effect of IFN-1 on different immunocytes in the chronic phase of HIV-1 infection. As corresponding authors, WK and YS carefully checked the whole manuscript.

## Conflict of Interest Statement

The authors declare that the research was conducted in the absence of any commercial or financial relationships that could be construed as a potential conflict of interest.

## References

[1] Barre-Sinoussi F Chermann JC Rey F Nugeyre MT Chamaret S Gruest J Isolation of a T-lymphotropic retrovirus from a patient at risk for acquired immune deficiency syndrome (AIDS). Science (1983) 220:868–71. 10.1126/science.6189183 6189183

[2] Martin AR Siliciano RF. Progress toward HIV eradication: case reports, current efforts, and the challenges associated with cure. Annu Rev Med (2016) 67:215–28. 10.1146/annurev-med-011514-023043 26526767

[3] Ruelas DS Greene WC. An integrated overview of HIV-1 latency. Cell (2013) 155:519–29. 10.1016/j.cell.2013.09.044 24243012 PMC4361081

[4] Rasaiyaah J Tan CP Fletcher AJ Price AJ Blondeau C Hilditch L HIV-1 evades innate immune recognition through specific cofactor recruitment. Nature (2013) 503:402–5. 10.1038/nature12769 24196705 PMC3928559

[5] Nissen SK Højen JF Andersen KL Kofod-Olsen E Berg RK Paludan SR Innate DNA sensing is impaired in HIV patients and IFI16 expression correlates with chronic immune activation. Clin Exp Immunol (2014) 177:295–309. 10.1111/cei.12317 24593816 PMC4089180

[6] Cunningham CR Champhekar A Tullius MV Dillon BJ Zhen A de la Fuente JR Type I and Type II interferon coordinately regulate suppressive dendritic cell fate and function during viral persistence. PLoS Pathog (2016) 12:e1005356. 10.1371/journal.ppat.1005356 26808628 PMC4726812

[7] Hardy MP Owczarek CM Jermiin LS Ejdeback M Hertzog PJ. Characterization of the type I interferon locus and identification of novel genes. Genomics (2004) 84:331–45. 10.1016/j.ygeno.2004.03.003 15233997

[8] Kotenko SV Gallagher G Baurin VV Lewis-Antes A Shen M Shah NK IFN-lambdas mediate antiviral protection through a distinct class II cytokine receptor complex. Nat Immunol (2003) 4:69–77. 10.1038/ni875 12483210

[9] Sandstrom TS Ranganath N Angel JB. Impairment of the type I interferon response by HIV-1: potential targets for HIV eradication. Cytokine Growth Factor Rev (2017) 37:1–16. 10.1016/j.cytogfr.2017.04.004 28455216

[10] Takeuchi O Akira S Pattern recognition receptors and inflammation. Cell (2010) 140:805–20. 10.1016/j.cell.2010.01.022 20303872

[11] Swaminathan S Sui H Adelsberger JW Chen Q Sneller M Migueles SA HIV-1 treated patients with undetectable viral loads have lower levels of innate immune responses via cytosolic DNA sensing systems compared with healthy uninfected controls. J AIDS Clin Res (2014) 5:315. 10.4172/2155-6113.1000315 26023356 PMC4444065

[12] Lee MN Roy M Ong SE Mertins P Villani AC Li W Identification of regulators of the innate immune response to cytosolic DNA and retroviral infection by an integrative approach. Nat Immunol (2013) 14:179–85. 10.1038/ni.2509 23263557 PMC3838897

[13] Lazear HM Lancaster A Wilkins C Suthar MS Huang A Vick SC IRF-3, IRF-5, and IRF-7 coordinately regulate the type I IFN response in myeloid dendritic cells downstream of MAVS signaling. PLoS Pathog (2013) 9:e1003118. 10.1371/journal.ppat.1003118 23300459 PMC3536698

[14] Iversen MB Ank N Melchjorsen J Paludan SR. Expression of type III interferon (IFN) in the vaginal mucosa is mediated primarily by dendritic cells and displays stronger dependence on NF-kappaB than type I IFNs. J Virol (2010) 84:4579–86. 10.1128/JVI.02591-09 20181703 PMC2863761

[15] Ranganath N Sandstrom TS Fadel S Cote SC Angel JB. Type I interferon responses are impaired in latently HIV infected cells. Retrovirology (2016) 13:66. 10.1186/s12977-016-0302-9 27613235 PMC5017046

[16] D’Ettorre G Paiardini M Ceccarelli G Silvestri G Vullo V. HIV-associated immune activation: from bench to bedside. AIDS Res Hum Retroviruses (2011) 27:355–64. 10.1089/aid.2010.0342 21309730

[17] Sandler NG Bosinger SE Estes JD Zhu RT Tharp GK Boritz E Type I interferon responses in rhesus macaques prevent SIV infection and slow disease progression. Nature (2014) 511:601–5. 10.1038/nature13554 25043006 PMC4418221

[18] Kløverpris HN Kazer SW Mjösberg J Mabuka JM Wellmann A Ndhlovu Z Innate lymphoid cells are depleted irreversibly during acute HIV-1 infection in the absence of viral suppression. Immunity (2016) 44(2):391–405. 10.1016/j.immuni.2016.01.006 26850658 PMC6836297

[19] Bosinger SE Li Q Gordon SN Klatt NR Duan L Xu L Global genomic analysis reveals rapid control of a robust innate response in SIV-infected sooty mangabeys. J Clin Invest (2009) 119:3556–72. 10.1172/JCI40115 19959874 PMC2786806

[20] Dragin L Nguyen LA Lahouassa H Sourisce A Kim B Ramirez BC Interferon block to HIV-1 transduction in macrophages despite SAMHD1 degradation and high deoxynucleoside triphosphates supply. Retrovirology (2013) 10:30. 10.1186/1742-4690-10-30 23497353 PMC3599726

[21] Harman AN Nasr N Feetham A Galoyan A Alshehri AA Rambukwelle D HIV blocks interferon induction in human dendritic cells and macrophages by dysregulation of TBK1. J Virol (2015) 89(13):6575–84. 10.1128/JVI.00889-15 25855743 PMC4468486

[22] Lahaye X Satoh T Gentili M Cerboni S Conrad C Hurbain I The capsids of HIV-1 and HIV-2 determine immune detection of the viral cDNA by the innate sensor cGAS in dendritic cells. Immunity (2013) 39(6):1132–42. 10.1016/j.immuni.2013.11.002 24269171

[23] Hrecka K Hao C Gierszewska M Swanson SK Kesik-Brodacka M Srivastava S Vpx relieves inhibition of HIV-1 infection of macrophages mediated by the SAMHD1 protein. Nature (2011) 474:658–61. 10.1038/nature10195 21720370 PMC3179858

[24] Beignon AS McKenna K Skoberne M Manches O DaSilva I Kavanagh DG Endocytosis of HIV-1 activates plasmacytoid dendritic cells via toll-like receptor-viral RNA interactions. J Clin Invest (2005) 115:3265–75. 10.1172/JCI26032 16224540 PMC1253628

[25] Saidi H Bras M Formaglio P Melki MT Charbit B Herbeuval JP HMGB1 is involved in IFN-alpha production and TRAIL expression by HIV-1-exposed plasmacytoid dendritic cells: impact of the crosstalk with NK cells. PLoS Pathog (2016) 12:e1005407 10.1371/journal.ppat.1005407 26871575 PMC4752468

[26] Beima-Sofie KM Bigham AW Lingappa JR Wamalwa D Mackelprang RD Bamshad MJ Toll-like receptor variants are associated with infant HIV-1 acquisition and peak plasma HIV-1 RNA level. AIDS (2013) 27(15):2431–9. 10.1097/QAD.0b013e3283629117 24037211 PMC4124859

[27] Bao M Liu YJ. Regulation of TLR7/9 signaling in plasmacytoid dendritic cells. Protein Cell (2013) 4:40–52. 10.1007/s13238-012-2104-8 23132256 PMC3667388

[28] Ewald SE Engel A Lee J Wang M Bogyo M Barton GM. Nucleic acid recognition by toll-like receptors is coupled to stepwise processing by cathepsins and asparagine endopeptidase. J Exp Med (2011) 208:643–51. 10.1084/jem.20100682 21402738 PMC3135342

[29] Liu MQ Zhao M Kong WH Tang L Wang F Zhu ZR Combination antiretroviral therapy (cART) restores HIV-1 infection-mediated impairment of JAK-STAT signaling pathway. Oncotarget (2017) 8(14):22524–33. 10.18632/oncotarget.15121 28186978 PMC5410242

[30] Liu L Botos I Wang Y Leonard JN Shiloach J Segal DM Structural basis of toll-like receptor 3 signaling with double-stranded RNA. Science (2008) 320:379–81. 10.1126/science.1155406 18420935 PMC2761030

[31] Bhargavan B Woollard SM Kanmogne GD Toll-like receptor-3 mediates HIV-1 transactivation via NFkappaB and JNK pathways and histone acetylation, but prolonged activation suppresses Tat and HIV-1 replication. Cell Signal (2016) 28:7–22. 10.1016/j.cellsig.2015.11.005 26569339 PMC4890564

[32] Verma R Bharti K. Toll like receptor 3 and viral infections of nervous system. J Neurol Sci (2017) 372:40–8. 10.1016/j.jns.2016.11.034 28017244

[33] Chattergoon MA Latanich R Quinn J Winter ME Buckheit RW Blankson JN HIV and HCV activate the inflammasome in monocytes and macrophages via endosomal toll-like receptors without induction of type 1 interferon. PLoS Pathog (2014) 10(5):e1004082. 10.1371/journal.ppat.1004082 24788318 PMC4006909

[34] Moody MA Santra S Vandergrift NA Sutherland LL Gurley TC Drinker MS Toll-like receptor 7/8 (TLR7/8) and TLR9 agonists cooperate to enhance HIV-1 envelope antibody responses in rhesus macaques. J Virol (2014) 88:3329–39. 10.1128/JVI.03309-13 24390332 PMC3957956

[35] Satoh T Kato H Kumagai Y Yoneyama M Sato S Matsushita K LGP2 is a positive regulator of RIG-I- and MDA5-mediated antiviral responses. Proc Natl Acad Sci U S A (2010) 107:1512–7. 10.1073/pnas.0912986107 20080593 PMC2824407

[36] Ebrahim M Mirzaei V Bidaki R Shabani Z Daneshvar H Karimi-Googheri M Are RIG-1 and MDA5 expressions associated with chronic HBV infection? Viral Immunol (2015) 28:504–8. 10.1089/vim.2015.0056 26485346

[37] Solis M Nakhaei P Jalalirad M Lacoste J Douville R Arguello M RIG-I-mediated antiviral signaling is inhibited in HIV-1 infection by a protease-mediated sequestration of RIG-I. J Virol (2011) 85(3):1224–36. 10.1128/JVI.01635-10 21084468 PMC3020501

[38] Kato H Takeuchi O Sato S Yoneyama M Yamamoto M Matsui K Differential roles of MDA5 and RIG-I helicases in the recognition of RNA viruses. Nature (2006) 441:101–5. 10.1038/nature04734 16625202

[39] Venkataraman T Valdes M Elsby R Kakuta S Caceres G Saijo S Loss of DExD/H box RNA helicase LGP2 manifests disparate antiviral responses. J Immunol (2007) 178:6444–55. 10.4049/jimmunol.178.10.6444 17475874

[40] Li P Kaiser P Lampiris HW Kim P Yukl SA Havlir DV Stimulating the RIG-I pathway to kill cells in the latent HIV reservoir following viral reactivation. Nat Med (2016) 22:807–11. 10.1038/nm.4124 27294875 PMC5004598

[41] Murphy TL Tussiwand R Murphy KM. Specificity through cooperation: BATF-IRF interactions control immune-regulatory networks. Nat Rev Immunol (2013) 13:499–509. 10.1038/nri3470 23787991

[42] Zhang M Liu Y Wang P Guan X He S Luo S HSV-2 immediate-early protein US1 inhibits IFN-beta production by suppressing association of IRF-3 with IFN-beta promoter. J Immunol (2015) 194:3102–15. 10.4049/jimmunol.1401538 25712217

[43] Gotoh K Tanaka Y Nishikimi A Nakamura R Yamada H Maeda N Selective control of type I IFN induction by the Rac activator DOCK2 during TLR-mediated plasmacytoid dendritic cell activation. J Exp Med (2010) 207:721–30. 10.1084/jem.20091776 20231379 PMC2856018

[44] Breckpot K Escors D Arce F Lopes L Karwacz K Van Lint S HIV-1 lentiviral vector immunogenicity is mediated by toll-like receptor 3 (TLR3) and TLR7. J Virol (2010) 84:5627–36. 10.1128/JVI.00014-10 20237085 PMC2876620

[45] Lee SM Suk K Lee WH. Synthetic peptides containing ITIM-like sequences of IREM-1 (CD300F) differentially regulate MyD88 and TRIF-mediated TLR signalling through activation of SHP and/or PI3K. Clin Exp Immunol (2012) 167:438–46. 10.1111/j.1365-2249.2011.04528.x 22288587 PMC3374276

[46] Zhao B Shu C Gao X Sankaran B Du F Shelton CL Structural basis for concerted recruitment and activation of IRF-3 by innate immune adaptor proteins. Proc Natl Acad Sci U S A (2016) 113:E3403–12. 10.1073/pnas.1603269113 27302953 PMC4914169

[47] Xu HG Liu L Gao S Jin R Ren W Zhou GP. Cloning and characterizing of the murine IRF-3 gene promoter region. Immunol Res (2016) 64:969–77. 10.1007/s12026-015-8780-8 26740329

[48] Vajjhala PR Ve T Bentham A Stacey KJ Kobe B. The molecular mechanisms of signaling by cooperative assembly formation in innate immunity pathways. Mol Immunol (2017) 86:23–37. 10.1016/j.molimm.2017.02.012 28249680

[49] Schlee M Hartmann G. Discriminating self from non-self in nucleic acid sensing. Nat Rev Immunol (2016) 16:566–80. 10.1038/nri.2016.78 27455396 PMC7097691

[50] Liu S Cai X Wu J Cong Q Chen X Li T Phosphorylation of innate immune adaptor proteins MAVS, STING, and TRIF induces IRF3 activation. Science (2015) 347(6227):a2630. 10.1126/science.aaa2630 25636800

[51] Gao D Wu J Wu YT Du F Aroh C Yan N Cyclic GMP-AMP synthase is an innate immune sensor of HIV and other retroviruses. Science (2013) 341:903–6. 10.1126/science.1240933 23929945 PMC3860819

[52] Jakobsen MR Bak RO Andersen A Berg RK Jensen SB Tengchuan J IFI16 senses DNA forms of the lentiviral replication cycle and controls HIV-1 replication. Proc Natl Acad Sci U S A (2013) 110:E4571–80. 10.1073/pnas.1311669110 24154727 PMC3845190

[53] Maelfait J Bridgeman A Benlahrech A Cursi C Rehwinkel J. Restriction by SAMHD1 limits cGAS/STING-dependent innate and adaptive immune responses to HIV-1. Cell Rep (2016) 16:1492–501. 10.1016/j.celrep.2016.07.002 27477283 PMC4978700

[54] Yoh SM Schneider M Seifried J Soonthornvacharin S Akleh RE Olivieri KC PQBP1 is a proximal sensor of the cGAS-dependent innate response to HIV-1. Cell (2015) 161:1293–305. 10.1016/j.cell.2015.04.050 26046437 PMC4503237

[55] Berg RK Rahbek SH Kofod-Olsen E Holm CK Melchjorsen J Jensen DG T cells detect intracellular DNA but fail to induce type I IFN responses: implications for restriction of HIV replication. PLoS One (2014) 9(1):e84513. 10.1371/journal.pone.0084513 24404168 PMC3880311

[56] Ysebrant DLL Martinet V Goriely S. Interferon regulatory factor 3 in adaptive immune responses. Cell Mol Life Sci (2014) 71:3873–83. 10.1007/s00018-014-1653-9 24879293 PMC11113752

[57] Jacquelin B Mayau V Targat B Liovat AS Kunkel D Petitjean G Nonpathogenic SIV infection of African green monkeys induces a strong but rapidly controlled type I IFN response. J Clin Invest (2009) 119(12):3544–55. 10.1172/JCI40093 19959873 PMC2786805

[58] Mandl JN Barry AP Vanderford TH Kozyr N Chavan R Klucking S Divergent TLR7 and TLR9 signaling and type I interferon production distinguish pathogenic and nonpathogenic AIDS virus infections. Nat Med (2008) 14(10):1077–87. 10.1038/nm.1871 18806803

[59] Del RI Bouchet J Alcover A. Studying the immune synapse in HIV-1 infection. Methods Mol Biol (2017) 1584:545–57. 10.1007/978-1-4939-6881-7_34 28255725

[60] Lavender KJ Gibbert K Peterson KE Van Dis E Francois S Woods T Interferon alpha subtype-specific suppression of HIV-1 infection in vivo. J Virol (2016) 90(13):6001–13. 10.1128/JVI.00451-16 27099312 PMC4907223

[61] Wilson EB Yamada DH Elsaesser H Herskovitz J Deng J Cheng G Blockade of chronic type I interferon signaling to control persistent LCMV infection. Science (2013) 340(6129):202–7. 10.1126/science.1235208 23580528 PMC3704950

[62] Audige A Hofer U Dittmer U van den Broek M Speck RF Evaluation of the immunomodulatory and antiviral effects of the cytokine combination IFN-alpha and IL-7 in the lymphocytic choriomeningitis virus and Friend retrovirus mouse infection models. Viral Immunol (2011) 24(5):375–85. 10.1089/vim.2011.0006 21929334

[63] Lee MS Park CH Jeong YH Kim YJ Ha SJ Negative regulation of type I IFN expression by OASL1 permits chronic viral infection and CD8(+) T-cell exhaustion. PLoS Pathog (2013) 9(7):e1003478 10.1371/journal.ppat.1003478 23874199 PMC3715418

[64] Park HJ Oh JH Ha SJ. Phenotypic and functional analysis of activated regulatory T cells isolated from chronic lymphocytic choriomeningitis virus-infected mice. J Vis Exp (2016) 112. 10.3791/54138 27404802 PMC4993242

[65] Lapierre P Janelle V Langlois MP Tarrab E Charpentier T Lamarre A. Expression of viral antigen by the liver leads to chronic infection through the generation of regulatory T cells. Cell Mol Gastroenterol Hepatol (2015) 1(3):325–41. 10.1016/j.jcmgh.2015.02.002 28210682 PMC5301191

[66] Lehmann C Lafferty M Garzino-Demo A Jung N Hartmann P Fätkenheuer G Plasmacytoid dendritic cells accumulate and secrete interferon alpha in lymph nodes of HIV-1 patients. PLoS One (2010) 5:e11110. 10.1371/journal.pone.0011110 20559432 PMC2885422

[67] Reeves RK Evans TI Gillis J Wong FE Kang G Li Q SIV infection induces accumulation of plasmacytoid dendritic cells in the gut mucosa. J Infect Dis (2012) 206:1462–8. 10.1093/infdis/jis408 22711907 PMC3529602

[68] Lehmann C Jung N Förster K Koch N Leifeld L Fischer J Longitudinal analysis of distribution and function of plasmacytoid dendritic cells in peripheral blood and gut mucosa of HIV infected patients. J Infect Dis (2014) 209(6):940–9. 10.1093/infdis/jit612 24259523

[69] Bruel T Dupuy S Démoulins T Rogez-Kreuz C Dutrieux J Corneau A Plasmacytoid dendritic cell dynamics tune interferon-alfa production in SIV-infected cynomolgus macaques. PLoS Pathog (2014) 10(1):e1003915. 10.1371/journal.ppat.1003915 24497833 PMC3907389

[70] Bloch N O’Brien M Norton TD Polsky SB Bhardwaj N Landau NR HIV type 1 infection of plasmacytoid and myeloid dendritic cells is restricted by high levels of SAMHD1 and cannot be counteracted by Vpx. AIDS Res Hum Retroviruses (2014) 30(2):195–203. 10.1089/AID.2013.0119 23924154 PMC3910455

[71] Kwa S Kannanganat S Nigam P Siddiqui M Shetty RD Armstrong W Plasmacytoid dendritic cells are recruited to the colorectum and contribute to immune activation during pathogenic SIV infection in rhesus macaques. Blood (2011) 118:2763–73. 10.1182/blood-2011-02-339515 21693759 PMC3172794

[72] O’Brien M Manches O Sabado RL Baranda SJ Wang Y Marie I Spatiotemporal trafficking of HIV in human plasmacytoid dendritic cells defines a persistently IFN-alpha-producing and partially matured phenotype. J Clin Invest (2011) 121:1088–101. 10.1182/blood-2011-02-339515 21339641 PMC3049388

[73] Calonge E Bermejo M Diez-Fuertes F Mangeot I González N Coiras M Different expression of interferon stimulated genes in response to HIV-1 infection in dendritic cells on their maturation state. J Virol (2017) 91:e1379–1316. 10.1128/JVI.01379-16 PMC5375683 28148784

[74] Balzarolo M Karrich JJ Engels S Blom B Medema JP Wolkers MC. The transcriptional regulator NAB2 reveals a two-step induction of TRAIL in activated plasmacytoid DCs. Eur J Immunol (2012) 42:3019–27. 10.1002/eji.201242385 22806638

[75] Manches O Fernandez MV Plumas J Chaperot L Bhardwaj N Activation of the noncanonical NF-kappaB pathway by HIV controls a dendritic cell immunoregulatory phenotype. Proc Natl Acad Sci U S A (2012) 109:14122–7. 10.1073/pnas.1204032109 22879398 PMC3435221

[76] Martin MP Qi Y Gao X Yamada E Martin JN Pereyra F Innate partnership of HLA-B and KIR3DL1 subtypes against HIV-1. Nat Genet (2007) 39:733–40. 10.1038/ng2035 17496894 PMC4135476

[77] Hölzemer A Thobakgale CF Jimenez Cruz CA Garcia-Beltran WF Carlson JM van Teijlingen NH Selection of an HLA-C*03:04-restricted HIV-1 p24 Gag sequence variant is associated with viral escape from KIR2DL3+ natural killer cells: data from an observational cohort in South Africa. PLoS Med (2015) 12:e1001900. 10.1371/journal.pmed.1001900 26575988 PMC4648589

[78] Haynes BF Gilbert PB McElrath MJ Zolla-Pazner S Tomaras GD Alam SM Immune-correlates analysis of an HIV-1 vaccine efficacy trial. N Engl J Med (2012) 366:1275–86. 10.1056/NEJMoa1113425 22475592 PMC3371689

[79] Kuri-Cervantes L de Oca GS Avila-Ríos S Hernández-Juan R Reyes-Terán G. Activation of NK cells is associated with HIV-1 disease progression. J Leukoc Biol (2014) 96(1):7–16. 10.1189/jlb.0913514 24399837

[80] Lichtfuss GF Cheng WJ Farsakoglu Y Paukovics G Rajasuriar R Velayudham P Virologically suppressed HIV patients show activation of NK cells and persistent innate immune activation. J Immunol (2012) 189(3):1491–9. 10.4049/jimmunol.1200458 22745371

[81] Jensen SS Fomsgaard A Borggren M Tingstedt JL Gerstoft J Kronborg G HIV-specific antibody-dependent cellular cytotoxicity (ADCC)-mediating antibodies decline while NK cell function increases during antiretroviral therapy (ART). PLoS One (2015) 10(12):e145249 10.1371/journal.pone.0145249 PMC4692281 26696395

[82] Martinez J Huang X Yang Y. Direct action of type I IFN on NK cells is required for their activation in response to vaccinia viral infection in vivo. J Immunol (2008) 180:1592–7. 10.4049/jimmunol.180.3.1592 18209055

[83] Beuneu H Deguine J Bouvier I Di Santo JP Albert ML Bousso P. Cutting edge: a dual role for type I IFNs during polyinosinic-polycytidylic acid-induced NK cell activation. J Immunol (2011) 187:2084–8. 10.4049/jimmunol.1004210 21810605

[84] Mizutani T Neugebauer N Putz EM Moritz N Simma O Zebedin-Brandl E Conditional IFNAR1 ablation reveals distinct requirements of type I IFN signaling for NK cell maturation and tumor surveillance. Oncoimmunology (2012) 1:1027–37. 10.4161/onci.21284 23170251 PMC3494617

[85] Naluyima P Eller MA Laeyendecker O Quinn TC Serwadda D Sewankambo NK Impaired natural killer cell responses are associated with loss of the highly activated NKG2A (+) CD57 (+)CD56(dim) subset in HIV-1 subtype D infection in Uganda. AIDS (2014) 28:1273–8. 10.1097/QAD.0000000000000286 24959961 PMC4032214

[86] Chung AW Isitman G Navis M Kramski M Center RJ Kent SJ Immune escape from HIV-specific antibody-dependent cellular cytotoxicity (ADCC) pressure. Proc Natl Acad Sci U S A (2011) 108:7505–10. 10.1073/pnas.1016048108 21502492 PMC3088575

[87] Waggoner SN Daniels KA Welsh RM. Therapeutic depletion of natural killer cells controls persistent infection. J Virol (2014) 88:1953–60. 10.1128/JVI.03002-13 24284324 PMC3911570

[88] Gotthardt D Sexl V STATs in NK-cells: the good, the bad, and the ugly. Front Immunol (2016) 7:694 10.3389/fimmu.2016.00694 28149296 PMC5241313

[89] Teijaro JR Ng C Lee AM Sullivan BM Sheehan KC Welch M Persistent LCMV infection is controlled by blockade of type I interferon signaling. Science (2013) 340:207–11. 10.1126/science.1235214 23580529 PMC3640797

[90] Serrano-Villar S Gutierrez C Vallejo A Hernández-Novoa B Díaz L Abad Fernández M The CD4/CD8 ratio in HIV-infected subjects is independently associated with T-cell activation despite long-term viral suppression. J Infect (2013) 66(1):57–66. 10.1016/j.jinf.2012.09.013 23046968

[91] Bastidas S Graw F Smith MZ Kuster H Günthard HF Oxenius A. CD8+ T cells are activated in an antigen-independent manner in HIV-infected individuals. J Immunol (2014) 192(4):1732–44. 10.4049/jimmunol.1302027 24446519

[92] Tough DF Borrow P Sprent J. Induction of bystander T cell proliferation by viruses and type I interferon in vivo. Science (1996) 272(5270):1947–50. 10.1126/science.272.5270.1947 8658169

[93] Kamath AT Sheasby CE Tough DF Dendritic cells and NK cells stimulate bystander T cell activation in response to TLR agonists through secretion of IFN-alpha beta and IFN-gamma. J Immunol (2005) 174(2):767–76. 10.4049/jimmunol.174.2.767 15634897

[94] Démoulins T Abdallah A Kettaf N Baron ML Gerarduzzi C Gauchat D Reversible blockade of thymic output: an inherent part of TLR ligand-mediated immune response. J Immunol (2008) 181:6757–69. 10.4049/jimmunol.181.10.6757 18981093

[95] Gorenec L Zidovec LS Grgic I Planinic A Iscic Bes J Vince A The comparison of Th1, Th2, Th9, Th17 and Th22 cytokine profiles in acute and chronic HIV-1 infection. Microb Pathog (2016) 97:125–30. 10.1016/j.micpath.2016.06.008 27268396

[96] Williams A Steffens F Reinecke C Meyer D. The Th1/Th2/Th17 cytokine profile of HIV-infected individuals: a multivariate cytokinomics approach. Cytokine (2013) 61:521–6. 10.1016/j.cyto.2012.11.006 23232337

[97] Clerici M Shearer GM A TH1→TH2 switch is a critical step in the etiology of HIV infection. Immunol Today (1993) 14:107–11. 10.1016/0167-5699(93)90208-3 8096699

[98] Herbeuval JP Grivel JC Boasso A Hardy AW Chougnet C Dolan MJ CD4+ T-cell death induced by infectious and noninfectious HIV-1: role of type 1 interferon-dependent, TRAIL/DR5-mediated apoptosis. Blood (2005) 106:3524–31. 10.1182/blood-2005-03-1243 16046522 PMC1895067

[99] Doitsh G Galloway NL Geng X Yang Z Monroe KM Zepeda O Cell death by pyroptosis drives CD4 T-cell depletion in HIV-1 infection. Nature (2014) 505:509–14. 10.1038/nature12940 24356306 PMC4047036

[100] Doitsh G Greene WC. Dissecting how CD4 T cells are lost during HIV infection. Cell Host Microbe (2016) 19:280–91. 10.1016/j.chom.2016.02.012 26962940 PMC4835240

[101] Barber DL Wherry EJ Masopust D Zhu B Allison JP Sharpe AH Restoring function in exhausted CD8 T cells during chronic viral infection. Nature (2006) 439:682–7. 10.1038/nature04444 16382236

[102] Crawford A Angelosanto JM Kao C Doering TA Odorizzi PM Barnett BE Molecular and transcriptional basis of CD4(+) T cell dysfunction during chronic infection. Immunity (2014) 40:289–302. 10.1016/j.immuni.2014.01.005 24530057 PMC3990591

[103] Cheng L Ma J Li J Li D Li G Li F Blocking type I interferon signaling enhances T cell recovery and reduces HIV-1 reservoirs. J Clin Invest (2017) 127:269–79. 10.1172/JCI90745 27941247 PMC5199717

[104] Perreau M Savoye AL De Crignis E Corpataux JM Cubas R Haddad EK Follicular helper T cells serve as the major CD4 T cell compartment for HIV-1 infection, replication, and production. J Exp Med (2013) 210:143–56. 10.1084/jem.20121932 23254284 PMC3549706

[105] Kohler SL Pham MN Folkvord JM Arends T Miller SM Miles B Germinal center T follicular helper cells are highly permissive to HIV-1 and alter their phenotype during virus replication. J Immunol (2016) 196:2711–22. 10.4049/jimmunol.1502174 26873986 PMC4779697

[106] Ray JP Marshall HD Laidlaw BJ Staron MM Kaech SM Craft J. Transcription factor STAT3 and type I interferons are corepressive insulators for differentiation of follicular helper and T helper 1 cells. Immunity (2014) 40:367–77. 10.1016/j.immuni.2014.02.005 24631156 PMC3992517

[107] Ma CS Avery DT Chan A Batten M Bustamante J Boisson-Dupuis S Functional STAT3 deficiency compromises the generation of human T follicular helper cells. Blood (2012) 119:3997–4008. 10.1182/blood-2011-11-392985 22403255 PMC3355712

[108] Lindqvist M van Lunzen J Soghoian DZ Kuhl BD Ranasinghe S Kranias G Expansion of HIV-specific T follicular helper cells in chronic HIV infection. J Clin Invest (2012) 122:3271–80. 10.1172/JCI64314 22922259 PMC3428098

[109] Buranapraditkun S Pissani F Teigler JE Schultz BT Alter G Marovich M Preservation of peripheral T follicular helper cell function in HIV controllers. J Virol (2017) 91:e497–417. 10.1128/JVI.00497-17 28468877 PMC5487582

[110] Abudulai LN Fernandez S Corscadden K Hunter M Kirkham LA Post JJ Chronic HIV-1 infection induces B-cell dysfunction that is incompletely resolved by long-term antiretroviral therapy. J Acquir Immune Defic Syndr (2016) 71(4):381–9. 10.1097/QAI.0000000000000869 26914910

[111] Sciaranghella G Tong N Mahan AE Suscovich TJ Alter G. Decoupling activation and exhaustion of B cells in spontaneous controllers of HIV infection. AIDS (2013) 27:175–80. 10.1097/QAD.0b013e32835bd1f0 23135171 PMC3729211

[112] Moir S Buckner CM Ho J Wang W Chen J Waldner AJ B cells in early and chronic HIV infection: evidence for preservation of immune function associated with early initiation of antiretroviral therapy. Blood (2010) 116:5571–9. 10.1182/blood-2010-05-285528 20837780 PMC3031405

[113] Rubtsov AV Rubtsova K Fischer A Meehan RT Gillis JZ Kappler JW Toll-like receptor 7 (TLR7)-driven accumulation of a novel CD11c(+) B-cell population is important for the development of autoimmunity. Blood (2011) 118:1305–15. 10.1182/blood-2011-01-331462 21543762 PMC3152497

[114] Moir S Fauci AS. Pathogenic mechanisms of B-lymphocyte dysfunction in HIV disease. J Allergy Clin Immunol (2008) 122:12–9. 10.1016/j.jaci.2008.04.034 18547629 PMC2708937

[115] Day CL Kaufmann DE Kiepiela P Brown JA Moodley ES Reddy S PD-1 expression on HIV-specific T cells is associated with T-cell exhaustion and disease progression. Nature (2006) 443:350–4. 10.1038/nature05115 16921384

[116] Kaufmann DE Kavanagh DG Pereyra F Zaunders JJ Mackey EW Miura T Upregulation of CTLA-4 by HIV-specific CD4+ T cells correlates with disease progression and defines a reversible immune dysfunction. Nat Immunol (2007) 8:1246–54. 10.1038/ni1515 17906628

[117] Gomez AM Ouellet M Tremblay MJ. HIV-1-triggered release of type I IFN by plasmacytoid dendritic cells induces BAFF production in monocytes. J Immunol (2015) 194:2300–8. 10.4049/jimmunol.1402147 25637018

[118] Zhang Z Cheng L Zhao J Li G Zhang L Chen W Plasmacytoid dendritic cells promote HIV-1-induced group 3 innate lymphoid cell depletion. J Clin Invest (2015) 125:3692–703. 10.1172/JCI82124 26301812 PMC4588300

[119] Chevalier MF Weiss L. The split personality of regulatory T cells in HIV infection. Blood (2013) 121:29–37. 10.1182/blood-2012-07-409755 23043072

[120] Pace L Vitale S Dettori B Palombi C La Sorsa V Belardelli F APC activation by IFN-alpha decreases regulatory T cell and enhances Th cell functions. J Immunol (2010) 184:5969–79. 10.4049/jimmunol.0900526 20427775

[121] Srivastava S Koch MA Pepper M Campbell DJ Type I interferons directly inhibit regulatory T cells to allow optimal antiviral T cell responses during acute LCMV infection. J Exp Med (2014) 211:961–74. 10.1084/jem.20131556 24711580 PMC4010906

[122] Barnaba V Schinzari V. Induction, control, and plasticity of Treg cells: the immune regulatory network revised? Eur J Immunol (2013) 43:318–22. 10.1002/eji.201243265 23408318

[123] Hori S Nomura T Sakaguchi S. Control of regulatory T cell development by the transcription factor Foxp3. Science (2003) 299:1057–61. 10.1126/science.1079490 12522256

[124] Fontenot JD Gavin MA Rudensky AY. Foxp3 programs the development and function of CD4+CD25+ regulatory T cells. Nat Immunol (2003) 4:330–6. 10.1038/ni904 12612578

[125] Pion M Jaramillo-Ruiz D Martinez A Munoz-Fernandez MA Correa-Rocha R. HIV infection of human regulatory T cells downregulates Foxp3 expression by increasing DNMT3b levels and DNA methylation in the FOXP3 gene. AIDS (2013) 27:2019–29. 10.1097/QAD.0b013e32836253fd 24201117

[126] Xie X Stubbington MJ Nissen JK Andersen KG Hebenstreit D Teichmann SA The regulatory T cell lineage factor Foxp3 regulates gene expression through several distinct mechanisms mostly independent of direct DNA binding. PLoS Genet (2015) 11:e1005251. 10.1371/journal.pgen.1005251 26107960 PMC4480970

[127] Moreno-Fernandez ME Rueda CM Rusie LK Chougnet CA Regulatory T cells control HIV replication in activated T cells through a cAMP-dependent mechanism. Blood (2011) 117:5372–80. 10.1182/blood-2010-12-323162 21436067 PMC3109711

[128] Lane HC Davey V Kovacs JA Interferon-alpha in patients with asymptomatic human immunodeficiency virus (HIV) infection. A randomized, placebo-controlled trial. Ann Intern Med (1990) 112:805–11. 10.7326/0003-4819-112-11-805 1971503

[129] Lane HC Kovacs JA Feinberg J Herpin B Davey V Walker R Anti-retroviral effects of interferon-alpha in AIDS-associated Kaposi’s sarcoma. Lancet (1988) 2:1218–22. 10.1016/S0140-6736(88)90811-2 2903954

[130] Angel JB Greaves W Long J Ward D Rodriguez AE Scevola D Virologic and immunologic activity of PegIntron in HIV disease. AIDS (2009) 23:2431–8. 10.1097/QAD.0b013e32832f30ca 19898218

[131] Tavel JA Huang CY Shen J Metcalf JA Dewar R Shah A Interferon-alpha produces significant decreases in HIV load. J Interferon Cytokine Res (2010) 30:461–4. 10.1089/jir.2009.0090 20235638 PMC2964361

[132] Asmuth DM Murphy RL Rosenkranz SL Lertora JJ Kottilil S Cramer Y Safety, tolerability, and mechanisms of antiretroviral activity of pegylated interferon Alfa-2a in HIV-1-monoinfected participants: a phase II clinical trial. J Infect Dis (2010) 201:1686–96. 10.1086/652420 20420510 PMC2946345

[133] Goujard C Emilie D Roussillon C Godot V Rouzioux C Venet A Continuous versus intermittent treatment strategies during primary HIV-1 infection: the randomized ANRS INTERPRIM Trial. AIDS (2012) 26:1895–905. 10.1097/QAD.0b013e32835844d9 22842994

[134] Boué F Reynes J Rouzioux C Emilie D Souala F Tubiana R Alpha interferon administration during structured interruptions of combination antiretroviral therapy in patients with chronic HIV-1 infection: INTERVAC ANRS 105 trial. AIDS (2011) 25:115–8. 10.1097/QAD.0b013e328340a1e7 20962614

[135] Azzoni L Foulkes AS Papasavvas E Mexas AM Lynn KM Mounzer K Pegylated Interferon alfa-2a monotherapy results in suppression of HIV type 1 replication and decreased cell-associated HIV DNA integration. J Infect Dis (2013) 207:213–22. 10.1093/infdis/jis663 23105144 PMC3532820

[136] Jiao YM Weng WJ Gao QS Hepatitis C therapy with interferon-alpha and ribavirin reduces the CD4 cell count and the total, 2LTR circular and integrated HIV-1 DNA in HIV/HCV co-infected patients. Antiviral Res (2015) 118:118–22. 10.1016/j.antiviral.2015.03.011 25823618

[137] Montes ML Nelson M Girard PM Sasadeusz J Horban A Grinsztejn B Telaprevir-based therapy in patients coinfected with chronic hepatitis C virus infection and HIV: INSIGHT study. J Antimicrob Chemother (2016) 71:244–50. 10.1093/jac/dkv323 26483516

[138] Sun H Buzon MJ Shaw A Hepatitis C therapy with interferon-alpha and ribavirin reduces CD4 T-cell-associated HIV-1 DNA in HIV-1/hepatitis C virus-coinfected patients. J Infect Dis (2014) 209:1315–20. 10.1093/infdis/jit628 24277743 PMC3982848

[139] Martinson JA Montoya CJ Usuga X Ronquillo R Landay AL Desai SN. Chloroquine modulates HIV-1-induced plasmacytoid dendritic cell alpha interferon: implication for T-cell activation. Antimicrob Agents Chemother (2010) 54:871–81. 10.1128/AAC.01246-09 19949061 PMC2812138

[140] Murray SM Down CM Boulware DR Stauffer WM Cavert WP Schacker TW Reduction of immune activation with chloroquine therapy during chronic HIV infection. J Virol (2010) 84:12082–6. 10.1128/JVI.01466-10 20844049 PMC2977889

[141] Paton NI Goodall RL Dunn DT Franzen S Collaco-Moraes Y Gazzard BG Effects of hydroxychloroquine on immune activation and disease progression among HIV-infected patients not receiving antiretroviral therapy: a randomized controlled trial. JAMA (2012) 308:353–61. 10.1001/jama.2012.6936 22820788 PMC3821003

[142] Routy JP Angel JB Patel M Kanagaratham C Radzioch D Kema I Assessment of chloroquine as a modulator of immune activation to improve CD4 recovery in immune nonresponding HIV-infected patients receiving antiretroviral therapy. HIV Med (2015) 16:48–56. 10.1111/hiv.12171 24889179

[143] Vaccari M Fenizia C Ma ZM Hryniewicz A Boasso A Doster MN Transient increase of interferon-stimulated genes and no clinical benefit by chloroquine treatment during acute simian immunodeficiency virus infection of macaques. AIDS Res Hum Retroviruses (2014) 30:355–62. 10.1089/AID.2013.0218 24251542 PMC3976588

[144] Zhen A Rezek V Youn C Lam B Chang N Rick J Targeting type I interferon-mediated activation restores immune function in chronic HIV infection. J Clin Invest (2017) 127:260–8. 10.1172/JCI89488 27941243 PMC5199686

[145] Moseman EA Wu T de la Torre JC Schwartzberg PL McGavern DB. Type I interferon suppresses virus-specific B cell responses by modulating CD8+ T cell differentiation. Sci Immunol (2016) 1:eaah3565. 10.1126/sciimmunol.aah3565 27812556 PMC5089817

